# Response to the Letter to the Editor Entitled “A Missing Genomic Dimension: The Small but Central Mitochondrial Genome in Diabetic Kidney Disease Genetics”

**DOI:** 10.1016/j.ekir.2026.106572

**Published:** 2026-04-29

**Authors:** Niina Sandholm, Joanne B. Cole, Viji Nair, Eoin Brennan, Elena Giardini, Jani K. Haukka, Eunji Ha, Anna Syreeni, Emma H. Dahlström, Rany M. Salem, Damian Fermin, Josep Mercader, Laura Smyth, Claire Hill, Josyf Mychaleckyj, Stuart McGurnaghan, Rachel G. Miller, Tina Costacou, Janet Snell-Bergeon, Andrew D. Paterson, Rasa Verkauskiene, Jelizaveta Sokolovska, Nicolae Mircea Panduru, Gianpaolo Zerbini, Kerstin Brismar, Andrzej S. Krolewski, Valma Harjutsalo, Peter Rossing, Samy Hadjadj, Gareth McKay, Amy Jayne McKnight, Alexander P. Maxwell, Katalin Susztak, Catherine Godson, Matthias Kretzler, Joel N. Hirschhorn, Jose C. Florez, Per-Henrik Groop, GENIE Consortium

**Affiliations:** 1 Folkhälsan Research Center, Helsinki, Finland; 2 Department of Nephrology, University of Helsinki and Helsinki University Hospital, Helsinki, Finland; 3 Faculty of Medicine, Research Program for Clinical and Molecular Metabolism, University of Helsinki, Helsinki, Finland; 4 Programs in Metabolism and Medical & Population Genetics, Broad Institute of Harvard and MIT, Cambridge, Massachusetts, USA; 5 Center for Genomic Medicine, Massachusetts General Hospital, Boston, Massachusetts, USA; 6 Department of Medicine, Harvard Medical School, Boston, Massachusetts, USA; 7 Division of Endocrinology, Boston Children’s Hospital, Boston, Massachusetts, USA; 8 Department of Biomedical Informatics, University of Colorado School of Medicine, Aurora, Colorado, USA; 9 Department of Medicine-Nephrology, University of Michigan School of Medicine, Ann Arbor, Michigan, USA; 10 Diabetes Complications Research Centre, School of Medicine and Conway Institute, University College Dublin, Dublin, Ireland; 11 Renal Electrolyte and Hypertension Division, Department of Medicine, University of Pennsylvania, Philadelphia, Pennsylvania, USA; 12 Institute of Diabetes Obesity and Metabolism, University of Pennsylvania, Philadelphia, Pennsylvania, USA; 13 Department of Genetics, University of Pennsylvania, Philadelphia, Pennsylvania, USA; 14 Herbert Wertheim School of Public Health and Human Longevity Science, University of California San Diego, La Jolla, California, USA; 15 Department of Pediatrics-Nephrology, University of Michigan School of Medicine, Ann Arbor, Michigan, USA; 16 Diabetes Section, Division of Endocrinology, Diabetes and Metabolism, Department of Medicine, Mass General Brigham, Boston, Massachusetts, USA; 17 Centre for Public Health, Queen’s University of Belfast, Belfast, UK; 18 Center for Public Health Genomics, University of Virginia, Charlottesville, Virginia, USA; 19 Western General Hospital, The Institute of Genetics and Cancer, University of Edinburgh, Edinburgh, UK; 20 Department of Epidemiology, School of Public Health, University of Pittsburgh, Pittsburgh, Pennsylvania, USA; 21 Barbara Davis Center for Diabetes, University of Colorado Denver, Aurora, Colorado, USA; 22 Genetics & Genome Biology Program, Research Institute, The Hospital for Sick Children, Toronto, Ontario, Canada; 23 Institute of Endocrinology, Lithuanian University of Health Sciences, Kaunas, Lithuania; 24 Faculty of Medicine and Life Sciences, University of Latvia, Riga, Latvia; 25 2nd Clinical Department, Carol Davila University of Medicine and Pharmacy, Bucharest, Romania; 26 Integrated Metabolic Investigation (IMI)—Center of Diabetes, Nutrition and Metabolism, Bucharest, Romania; 27 Complications of Diabetes Unit, Division of Immunology, Transplantation and Infectious Diseases, Diabetes Research Institute, IRCCS San Raffaele Scientific Institute, Milano, Italy; 28 Department of Molecular Medicine and Surgery, Rolf Luft Center for Diabetes Research and Endocrinology, Karolinska Institutet, Stockholm, Sweden; 29 Department of Endocrinology, Diabetes and Metabolism, Karolinska University Hospital, Stockholm, Sweden; 30 Section on Genetics and Epidemiology, Research Division, Joslin Diabetes Center, Boston, Massachusetts, USA; 31 Steno Diabetes Center Copenhagen, Herlev, Denmark; 32 Department of Clinical Medicine, University of Copenhagen, Copenhagen, Denmark; 33 INSERM CIC (Centre d'Investigation Clinique) 1402 and U 1082 (IRTOMIT), Poitiers, France; 34 Department of Endocrinology, L'institut du thorax, INSERM, Centre National de la Recherche Scientifique, Centre Hospitalier Universitaire de Nantes, Nantes, France; 35 Regional Nephrology Unit, Belfast City Hospital, Belfast, UK; 36 Penn-CHOP Kidney Innovation Center, University of Pennsylvania, Philadelphia, Pennsylvania, USA; 37 Department of Internal Medicine, University of Michigan, Ann Arbor, Michigan, USA; 38 Department of Pediatrics and Genetics, Harvard Medical School, Boston, Massachusetts, USA; 39 Department of Diabetes, Central Clinical School, Monash University, Melbourne, Victoria, Australia; 40 Baker Heart and Diabetes Institute, Melbourne, Victoria, Australia

**The Author Replies:**

Recent genetic investigations, especially genome-wide association studies have identified multiple common genetic factors associated with diabetic kidney disease (DKD).^1^^,^^2^ To focus on the role of low-frequency and rare variation for DKD risk, we conducted exome-wide analysis based on exome-array data for 10,312 individuals with type 1 diabetes, and identified a missense variant (p.Asp555Asn) in *EXD3* associated with DKD.^3^ Furthermore, gene-level analyses identified multiple low-frequency missense variants in *MUC5B* associated with estimated glomerular filtration rate-based phenotypes. Although whole-exome or whole-genome sequencing would provide deeper rare variant detection, our exome array approach provided a cost-effective compromise to increase statistical power and address low-frequency variants.

In their letter to the Editor, Dr. Ural^4^ highlights the role of mitochondrial DNA (mtDNA) sequence variation, heteroplasmy, and mtDNA copy number, which have been associated with DKD. This point is further supported by the proposed role of mitochondrial energetics in diabetes and its complications. Although mtDNA can be partly captured by genome-wide association studies genotyping arrays, whole-genome sequencing and targeted quantitative polymerase chain reaction assays are better suited to assess mtDNA variation. In contrast to autosomal single-nucleotide polymorphism variation, mtDNA copy number may vary by tissue and change in response to DKD, and therefore an association does not imply causality. Furthermore, the majority (∼99%) of the mitochondrial proteome is encoded by nuclear DNA, whose coding variation is largely captured by our approach. Nevertheless, we agree that mitochondrial function represents a biologically relevant domain of DKD research. Although out of scope of our exome-analysis,^3^ ongoing efforts aim to clarify the role of mtDNA sequence and copy number variation in DKD.^5^

Furthermore relevant genetic variation includes copy number and structural variation, and somatic changes, such as mosaic loss of chromosomes and clonal hematopoiesis of indeterminate potential. Epigenetic DNA variation, including telomere length and DNA methylation, is also associated with DKD risk. Versatile genetics approaches including larger genome-wide association studies and whole-genome sequencing are needed, combined with epigenetic analysis, comprehensive omics integration, and functional validation to truly understand the genetic background of DKD. The ultimate aim is to better predict DKD risk and delineate causal biology to enable more personalized treatments that can prevent the onset and progression of DKD.
